# World Hepatitis Day — July 28, 2015

**Published:** 2015-07-24

July 28, 2015, marks the fifth annual World Hepatitis Day, established in 2010 by the World Health Organization to increase awareness and understanding of viral hepatitis. Millions of acute hepatitis infections occur each year, and approximately 400 million persons are living with chronic hepatitis B or hepatitis C (1). An estimated 1.4 million persons die each year from the various forms of viral hepatitis (1). The theme of this year’s World Hepatitis Day is “Prevent Hepatitis. Act Now.” Key messages will focus on risks, safe injection practices, vaccination, and testing and treatment.

This issue of *MMWR* includes a report describing the launch of a nationwide hepatitis C elimination program in Georgia, a country with a high burden of hepatitis C. The initial phase of the program is focused on increasing access to affordable diagnostics, free treatment of persons with severe liver disease who are at highest risk for hepatitis C–related morbidity and mortality with new curative regimens, and building capacity to achieve program goals of prevention of transmission and elimination of disease. Georgia’s program might provide information and experience that can inform similar efforts in other parts of the world.

A second report summarizes viral hepatitis surveillance and outbreak data from a national surveillance system in India for epidemic-prone diseases. This report sheds light on the burden and epidemiology of acute viral hepatitis in India, particularly hepatitis A and E, and highlights the important role that routine hepatitis surveillance can play in guiding prevention efforts.

Additional information about World Hepatitis Day is available at http://worldhepatitisday.org. Resources for health professionals are available at http://www.cdc.gov/hepatitis.
